# Mental Health Services for Adolescents With Status as Asylum Seekers or Refugees: A Qualitative Study of Healthcare Professionals' Perspectives

**DOI:** 10.1177/13634615251409677

**Published:** 2026-01-09

**Authors:** Petter Viksveen, Lobna Doudouh, Murad Mustafa Jafaer, Anita Salamonsen

**Affiliations:** 1 Department for Quality and Health Technology, SHARE - Centre for Resilience in Healthcare, 621781 University of Stavanger, Norway; 2 Department of Housing and Care, Abri Dialogue, Sola, Norway; 3 Regional Centre for Child and Youth Mental Health and Child Welfare - North (RKBU North), 60482 UiT The Arctic University of Norway, Tromsø, Norway

**Keywords:** mental health services, adolescents, asylum seekers and refugees, qualitative study

## Abstract

Clear recommendations for how to best adapt mental health services for adolescents with a background as asylum seekers and refugees are lacking. This study therefore explored healthcare professionals’ experiences and perspectives on mental health needs of these groups of youth in Norway. The objectives were to explore healthcare professionals’ perspectives on what is needed for adolescents with status as asylum seekers or refugees to seek professional help for mental health problems and what is important for them to experience the offered help as beneficial. A qualitative interview study was carried out to explore the perspectives of healthcare professionals (*n* = 11) in primary and secondary healthcare settings. Systematic text condensation was used for data analysis. Four themes were developed through the study: (a) culturally sensitive and adapted services; (b) low threshold and outreach services; (c) building a trusting relationship; and (d) user involvement. The study provides a constructivist understanding of cultural competence including several suggestions for how to overcome barriers to service use among adolescents who have a background as asylum seekers or refugees. Some of the recommendations include an ongoing collaboration with youth and their families to better understand their culture; healthcare professionals who acquire cultural competence, practice cultural humility and are mindful of their own cultural backgrounds. There should be comprehensive education and training programmes for healthcare professionals. Services should be easily accessible with simplified referral procedures and arena flexibility. Skilled interpreters should be used, including cultural interpreters. Shared decision-making should be used to actively engage youth in their treatment.

## Introduction

More than 100 million people worldwide have been forcibly displaced, according to the United Nations High Commissioner for Refugees (UNHCR, 2023a). Unlike voluntary migration motivated by work or education, forced displacement includes internally displaced persons (IDPs) or refugees. Both groups are forced to leave their homes, but IDPs move within their country's borders ‘to avoid the effects of armed conflict, situations of generalized violence, violations of human rights or natural or human-made disasters’, whereas refugees cross borders because of a ‘well-founded fear of being persecuted for reasons of race, religion, nationality, membership of a particular social group or political opinion’. There are about 35 million refugees worldwide, of whom half are children or adolescents (UNHCR, 2023a). Young refugees may be accompanied by family or travel alone, classified as unaccompanied minors. Asylum seekers await legal permission to stay or face return to their country of origin. In 2021, 27,000 unaccompanied children applied for asylum, a 28% increase compared to 2020. The war in Ukraine has become the fastest-growing refugee emergency with 6.3 million displaced (UNHCR, 2023b), of whom 40% are children (Brass & Greenberg, 2022). Moreover, there are about two million Palestinian refugees or internally displaced across Gaza, the West Bank, and the surrounding areas (Palestinians, 2024). Climate change is expected to significantly increase forced migration in the coming years (Kronick et al., 2021).

According to UNHCR (2022), ‘[…] children, adolescents and youth are at risk of various forms of abuse, separation from their carers, neglect, violence, exploitation, trafficking or military recruitment’. Together with trauma from war, uncertainty about refugee status, resettlement stress, and low socio-economic status, these factors increase the risk of mental health disorders (Giacco et al., 2014; Priebe et al., 2016). Adolescent refugees and asylum seekers have poorer mental health and higher rates of mental disorders than the general population of adolescents (Beiser & Hou, 2017; Blackmore et al., 2020). Reported prevalence rates include anxiety (16–38%), depression (14–44%), and post-traumatic stress disorder (PTSD) (23–54%) (Blackmore et al., 2020; Bronstein et al., 2013; Jensen et al., 2015; Vervliet et al., 2014a). These problems persist over time after arrival to a host country (Jensen et al., 2014; Priebe et al., 2016; Vervliet et al., 2014b), even over decades (Vaage et al., 2010).

International surveys reveal that youth with refugee backgrounds are less likely to use mental health services, compared to the majority youth population (Berg et al., 2021; De Montgomery et al., 2020; Gubi et al., 2022; Kamali et al., 2023). For example, most asylum seekers and refugees from the Middle East do not seek help for conditions such as anxiety, depression and PTSD (Boettcher et al., 2021; Fuhr et al., 2019). However, service use varies within refugee groups. Youth granted asylum tend to access services more often than those reunited with family (De Montgomery et al., 2020). Barriers to mental health service use among refugees from Africa, Asia, Latin America, the Middle East, and the West Indies include structural factors such as cost, long wait times, language barriers, and limited cultural competence among health providers (Boettcher et al., 2021; Fauk et al., 2021; Salam et al., 2022; Salami et al., 2019). Social factors such as stigma, cultural perceptions of mental health, and poor health literacy further hinder access.

A recent systematic review suggests that both migrant service users (including refugees) and mental health providers emphasize the importance of linguistically and culturally appropriate care (Krystallidou et al., 2024). Providers face communication challenges when discussing mental health, describing the available public services and practitioners’ roles with service users. Beyond common issues like insufficient time and funding, barriers to service use include language difficulties and insufficient knowledge about service users’ cultural backgrounds. Healthcare professionals lack cultural awareness and competence, and training to improve their knowledge, and access to professional interpreters seems inadequate.

The UN Declaration of Human Rights emphasizes everyone's right to an adequate standard of living, including good health and access to care. In 2016, the World Psychiatry Association urged a global humanitarian agenda to ensure refugees’ and asylum seekers’ right to proper mental health services. Others have stressed the importance of improving reception and care facilities for refugee youth (Vervliet, Lammertyn, et al., 2014), and improvements of mental health systems for refugees (Bäärnhielm et al., 2017). Although general suggestions such as screening and adapted psychosocial care exist, clear suggestions for the best way to adapt mental health services to meet the mental health needs of youth are lacking (Healey et al., 2017).

This study aimed to analyse health professionals’ perspectives on mental health service needs of asylum-seeker and refugee adolescents in Norway. Adolescents are in the Norwegian health system defined as those aged 12 to 18, although some continue therapy beyond 18. Scandinavian welfare states such as Norway in principle guarantee asylum seekers and refugees access to high-quality mental health services through the public health system (Patient and User Rights Act, 2021). All municipalities offer mental health services for the general public, including children and adolescents (Helsenorge, 2023), though service delivery varies. Mental health pathways were established in 2019 to further ensure the rights for timely and effective assessment and treatment (Norwegian Government, 2021). However, research-based knowledge providing insight into the challenges and potential solutions for these adolescents are lacking.

The objectives of this study were to explore healthcare professionals’ perspectives on what it takes for adolescents with status as asylum seekers or refugees to seek professional help for mental health problems and what healthcare professionals consider important for these groups of adolescents to feel they are helped.

## Methods

### Design, Recruitment and Participants

A qualitative phenomenological interview study (Järvinen & Mik-Meyer, 2020) with open-ended questions was conducted to explore the perspectives of healthcare professionals in Norway's primary and secondary care. This is appropriate when studying experience-based, underexplored topics (Järvinen & Mik-Meyer, 2020). The term ‘healthcare professional’ refers to trained, publicly regulated individuals, distinguished from ‘health personnel’, which includes any person involved in health services, regardless of training or regulation (MeSH term D006282).

Purposeful sampling was used to recruit participants (Patton, 1990), using maximum variation sampling to capture different perspectives among healthcare professionals. This included practitioners treating the general population and working solely with immigrants and refugees, across general practitioner (GP) clinics, municipal nursing services, and hospital-based health services, in two municipalities. Interested healthcare professionals received study information and consent forms. All but one agreed to participate.

All participants had experience treating asylum-seeker or refugee adolescents with mental health problems. Adolescents in Norway first contact primary care services and most are not referred for secondary/specialist care. Therefore, most participants (8 of 11) came from primary care, including school nurses (*n* = 6) and GPs (*n* = 2). The remaining three participants were from secondary care: a psychiatrist, a psychologist, and a clinical social worker. Most had extensive experience treating these adolescents, some for more than 10 years, including working in reception centres or refugee camps abroad. All had used interpreters during consultations. Most were female (*n* = 10), and aged primarily from 40 to 50 years (*n* = 6). The youngest was in her 30s and the oldest was over 60 years.

The study aims for sufficient information power rather than data saturation. Information power depends on specificity of the sample, dialogue quality, and relevance to the research questions (Malterud et al., 2016). The sample of 11 participants was considered sufficient.

### Data Collection

All interviews were carried out by one of two researchers (LD, PV) using a semi-structured guide (Appendix A). Interviews took place in-person at locations chosen by the participants, mostly in their clinics, except one who was interviewed at the research centre. Interviewers and interviewees had not met before. Interviews were audio-recorded and transcribed verbatim. An interview guide with two key questions was used: ‘What do you think is important in order for adolescents with status as asylum seekers or refugees to seek professional help for mental health conditions and challenges?’ and ‘What do you think is important in order for adolescents to feel they benefit from the help?’. Participants were given time to reflect, elaborate and share examples, for deeper insight into their experiences and perspectives. Their reasoning was explored to gain a deeper understanding (Wengraf, 2001). The interviews lasted for an average of 54 minutes (median 50, interquartile range 34–71).

### Data Analysis

Systematic text condensation (STC) was used for data analysis (Malterud, 2012), based on Giorgi's inductive, data-driven, descriptive phenomenological method (Järvinen & Mik-Meyer, 2020). It was used to describe healthcare professionals’ experiences and perspectives, and included a four-stage process: (a) identification of preliminary themes; (b) development of code groups; (c) development of subgroups and condensation of subgroups (with citations); and (d) data synthesis.

The analysis was conducted by two researchers (PV, AS) and checked by the third researcher (LD) and the co-researcher (MMJ). The 120 pages of transcripts were revisited several times to ensure accurate representation of participants’ experiences and perspectives. In stage one, transcripts were independently read to identify preliminary themes. Stage two involved coding solely based on participants’ statements, making the analysis data-driven. In stage three, subgroups were formed and data condensed with supporting citations. In stage four we synthesized the content into themes, presented with participant quotes. Table 1 illustrates the analytical process.

**Table 1.** Example of the Analysis Using Systematic Text Condensation.

Preliminary theme	Code group	Subgroups	Condensate	Citation
Culturally sensitive services	Mental health problems understood differently	1. Language 2. Culture 3. Gender	Healthcare professionals must have cultural understanding, to be culturally sensitive to understand, reach and treat these adolescents	How do I approach this boy, man, girl, woman? Can I shake their hands? Not always. I need to understand why she is holding her hand back

In the discussion following the results section, we further reflect on themes identified in the STC.

### Researchers' Backgrounds, and Public Involvement

The research team included two experienced researchers (PV and AS), a master's student (LD) and a youth co-researcher (MMJ). PV is an associate professor at SHARE – Centre for Resilience in Healthcare in the Faculty of Health Sciences at the University of Stavanger. Since 2017 he has lead youth mental health research, with recent focus on minority youth. LD conducted the study as part of her Master of Drug and Mental Health Work, supervised by PV. Originally from Morocco, she has experience from youth care and currently leads a private care service team leader for a private company providing services in a range of areas including housing and care services for children, people with disabilities, mental health and substance problems. MMJ, a former refugee currently working as a nurse in Norwegian psychiatric care, has contributed to PV's projects since 2018. He has helped plan research projects, collected and analysed data, presented results at conferences, and co-authored scientific articles. He gave input to the interview guide, focus of interviews, made comments to the analysis, and co-authored this article. AS is a sociology professor at The Regional Centre for Child and Youth Mental Health and Child Welfare, North (RKBU North) at UiT The Arctic University of Norway, and Professor II in sociology at SHARE. Her research focuses on youth involvement in mental health and welfare, and services for Indigenous, national minority and refugee youth.

## Results

Four themes were developed to address adolescents’ access to and benefit of services: (a) culturally sensitive and adapted services; (b) low threshold and outreach services; (c) building a trusting relationship; and (d) user involvement.

### Culturally Sensitive and Adapted Services

Healthcare professionals described cultural background as significant to how adolescents perceive mental health and services. They emphasized the importance of culturally sensitive and adapted services to understand, reach and treat these adolescents.

Awareness of cultural background was important to understand adolescents’ perspectives and behaviours. Some were not familiar with concepts like mental health, psychology and psychiatry: ‘Western psychiatry is a cultural invention. And I’m trained to practise it and believe in it in a way, but I cannot take for granted that this understanding is valid all around the world’ (P7). Mental health was associated with considerable stigma, and mental health problems were understood differently from the Norwegian majority population: ‘[They are] not used to consulting with a doctor and say that: “I’m depressed or I struggle with my thoughts”, because you’re crazy then’ (P8), and ‘[…] mental health is not part of their vocabulary. […] they don’t know that there is any such thing as mental health’ (P11). Some adolescents would ascribe symptoms of mental health to entirely different causes, not uncommonly associated with so-called jinns or spirits: ‘They all have different backgrounds and we might think that everyone thinks like we do in Norway, but they don’t. […] it can be much more extreme, demons and such things […]’ (P1).

Understanding cultural backgrounds helped health services reach adolescents. Many adolescents turn to family members, rather than professionals: “Many countries do not even have this type of public service. […] they seek help from family members or others, and they don’t really talk much about it at all’ (P4). Sharing mental health information via parents, other trusted relatives or schools could help to reach adolescents with asylum-seeker or refugee backgrounds. This was important, because many adolescents had poor social networks, thereby limiting the number of trusted information sources. Information could include who to seek help from, such as school nurses and GPs, or specialist mental health professionals. Practical information like clinic location and opening hours were often difficult for these adolescents to access.

Cultural insight helped healthcare professionals build trust and establish good dialogue with adolescents, including knowing how to greet them and what to expect during consultations.

How do I approach this boy, man, girl, woman? Can I shake their hands? Not always. I need to understand why she is holding her hand back. Can I ask to see her face? […] There may be cultural, religious reasons why you can’t. (P1)

Adolescents’ cultural backgrounds could also affect what healthcare professionals could talk about and when to do so. ‘They might not be used to you going right at it, but rather talk about different things. […] you might get more to the core of the problem towards the end of the conversation’ (P4). For many adolescents, building trust takes more time. Cultural insight helped healthcare professionals understand adolescents’ emotions, such as cultures where crying was seen as harmful, as adolescents believed it could cause somatic complaints or ‘open up for evil to enter’ (P7).

Healthcare professionals acknowledged they could not fully prepare for the broad spectrum of cultural backgrounds and therefore the different perceptions of mental health. Adolescents’ educational level could vary considerably, contributing to different understandings of mental health, even within one ethnic group. Therefore, healthcare professionals first expressed interest in adolescents’ cultural backgrounds, gained insight into their backgrounds and understanding of mental health and services. Professionals should reflect upon and become aware of their perceptions in light of their own cultural background:

[…] be curious, be interested in other cultures, and recognise that I have my own cultural glasses on. To be Norwegian and white does not mean that I’m neutral and that it's others who have a culture and who do things differently. […] Which culture do I come from? How do I understand the world? And then be open to: Who are you? Where do you come from? What affects you and your perception of life? (P7)

Curiosity about adolescents’ cultural background was considered essential, but not sufficient. A more systematic and systemic approach was required to strengthen cultural competence, as most had minimal training: ‘There was a little bit in the course for school nurses. I thought this was a bit of a “side-issue” then, but I should have known much more before starting to practise’ (P4). Some suggested extensive training to build cultural competence, focusing on cultural sensitivity, migration health, human rights perspectives, reactions to war and trauma, sexuality, and learning how to use an interpreter.

This is not just for those who have a particular interest. Norway has become a society where everyone, to a larger or smaller extent, must relate to a much more heterogeneous population, people with very different backgrounds. We can’t get stuck in stereotypical thinking. The typical idea now is with Muslims, it's as if that defines the entire person. But they have many other identities, as parents, in sports, work life, anything that we all have that identifies us. We need to move away from us–them ways of thinking. (P9)

Finally, to better understand asylum-seeking and refugee adolescents, all participants highlighted the importance of interpreters to manage language barriers. Adolescents’ willingness to use interpreters also depended on their cultural background. Although some youth felt comfortable with interpreters, others were reluctant because they feared or expected information could be passed on to their community or even the government in their country of origin. Healthcare professionals offered flexible options for interpreter use, in-person or by telephone. Cultural interpreters could help to better understand and communicate with adolescents. Cultural interpreters are familiar with adolescents’ culture, and the majority culture, and help to understand the meaning of communicated information (Abdi et al., 2020; Gómez & Pinazo, 2018).

A cultural interpreter tries to help to clarify phenomena. They may have understandings of disease and health […] which for us who come from a Norwegian context don’t automatically understand, and which can be helpful to discuss […] Is this due to culture or is it an expression of disease? (P8)

### Low Threshold and Outreach Services

Healthcare professionals considered low threshold and outreach services important for adolescents to get in touch. Gender-specific approaches for service access were particularly important for girls, because they were less likely to seek help.

Information about the services and how adolescents could contact healthcare professionals should be easily available and provided through multiple public channels: ‘[…] information which may come from their support networks, parents, schools, health station, to let them know the GP is there to help’ (P2), and ‘It's important to repeat the information on different arenas and share it, make it easily available. School nurses inform about it in upper secondary school, introduction classes for immigrants and language learning centres’ (P8).

Flexible referral pathways were suggested to access both primary care (e.g. school nurses, GPs), and specialist mental health services (e.g. psychologists, psychiatrists, within inpatient and outpatient care). Although GPs may serve as a ‘point of entry’, specialist care should also be more open for direct contact, especially for newcomers:

Typical for secondary health services is a requirement for referral from a GP. […] But that should not determine if they come […] it's an additional threshold. They should be able to be referred from primary care, learning centres, refugee health services, schools, and patients themselves. (P9)

Current legislation required healthcare professionals to involve parents of adolescents under the age of 16. This introduced an additional barrier to adolescents’ service use, as described by a school nurse who recommended a simplified referral procedure: ‘[…] under the age of 16, if it could be possible to not have to call the parents. [To] get the parents to contact the GP, and the GP to refer, that's a big leap’ (P10).

Booking systems should be simple to understand, easy to find, and with flexible opening hours. Services should be free and offer more than the standard ten sessions for mental health assessment and treatment (Norwegian Directorate of Health, 2020). It was suggested to extend service access up to age 25. Currently, youth aged 18–25 attending adult primary school could only access health services located far away from their schools: ‘They do have access to the health station for youth, but they don’t, because they don’t understand what it is. Those who attend primary school for adults don’t use the health station for youth’ (P10).

Flexible outreach services could support adolescents, especially those reluctant to seek help. Healthcare professionals were encouraged to provide outreach activities in adolescents’ own arenas, including schools, workplaces, football fields or at home:

You can’t expect them to come where you are. You need to move out of your office […] The health station for youth […] with arena flexibility, they could come to the school [were] they have mental and sexual health competence. (P10)

### Building a Trusting Relationship

Healthcare professionals emphasized building trust for adolescents to feel confident in and use the services. Owing to cultural background and past trauma, many felt uncertain about who they could trust. This required professionals to spend considerable time to gradually build the relationship: ‘We often spend considerable time to establish trust and contact. […] Being a refugee is to have developed an ability not to trust people […] it's a necessary survival strategy’ (P7).

These adolescents need professionals who are understanding and present for them. This was achieved by showing interest and expressing humility; listening attentively to adolescents’ stories; and expressing their understanding: ‘We need to be willing to meet the persons, listen to their stories and thoughts about the world. Humility with regards to others who may have lots of good in their culture that we could learn from’ (P4). Healthcare professionals with a refugee or immigrant background could share own experiences and express understanding. The process of establishing trust involved meeting adolescents’ information needs, especially explaining and consistently upholding confidentiality. It also meant not necessarily focusing on mental health and trauma, but on issues of interest to adolescents including daily life activities, or issues with partners or sexuality. Healthcare professionals should let adolescents pose questions. However, healthcare professionals described that for some adolescents it was also important that they appeared as competent professionals: ‘Many want me as a doctor to be more determined’ (P7). Moreover, healthcare professionals should express willingness to help, and be ready to provide advice and practical support:

Talking a lot and digging too much can contribute to retraumatising. Very often those everyday things, getting started, leisure time activities, finance, just help a little bit with this can be of just as much help as sitting and talking. (P5)

Trust should be developed through the continuity of reliable support; for example, through regular follow-up consultations over time and referral to specialists when needed. It could require exceeding the standard limit of ten consultations in secondary mental health services. If not, ongoing care might be better provided through primary care services.

### User Involvement

Healthcare professionals considered user involvement important to provide adolescents with opportunities to participate actively to plan, carry out and assess treatment results, and to develop ownership of the therapeutic process: ‘User involvement is important, otherwise you won’t manage anything. […] to help them feel it's “their” treatment’ (P3).

Adolescents should be involved in planning and adapting treatment to meet their needs. This could be facilitated by focusing on their needs, letting them decide the focus of conversations, taking them seriously, and treating them respectfully. Active participation could be facilitated by highlighting their strengths, focusing on their opportunities and providing them with questions for reflection: ‘I often challenge them, so they start reflecting about things. I can give them assignments, think about it. […] where do you want to be in one year, in life? […] this is a form of user involvement’ (P1).

Some adolescents might become actively involved in dialogue with ‘like-minded’ peers. They could easily understand each others’ challenges and relate to feeling uncertain about treatment and the services:

I think those who have experience can help those who are currently struggling. […] Instead of us therapists spending thousands of hours to convey it to them, it's better for them to hear it from adolescents who are around the same age and who have the same ethnic background (P6).

Healthcare professionals should use shared decision-making in treatment, providing adolescents with information to develop and express their opinion. They should be asked about their preferences and their opinions should influence decisions: ‘You need shared decision-making, determine whether they agree about the focus of treatment or if they want to raise other issues’ (P3). However, some also expressed doubts and they thought it should sometimes be balanced with mild paternalism.

They didn’t come to me, I went to them. I talk a bit first […] and withdraw when I’ve talked enough. […] I know they might benefit from something that they don’t even know exists. I can’t tell them you must be struggling mentally now and at the same time let them decide. User involvement is quite tricky, a challenging concept really. It sounds all natural, but what is it really and to what extent? (P10)

User involvement could also encourage adolescents to use their own resources and manage things by themselves. This could for example be achieved by motivating them to pursue education, build competence, boost self-confidence, expand social networks and job prospects, and support their mental health.

## Discussion

This was the first Norwegian study exploring healthcare professionals’ perspectives on how adolescents with asylum-seeker or refugee status seek professional help for mental health problems and what makes them feel supported. Interviews with primary and secondary healthcare professionals resulted in four themes for improving the services: cultural sensitivity and adaptation to adolescents’ needs; active involvement of adolescents in their care; low threshold easily accessible services; and development of a trusting relationship.

Healthcare professionals in this study emphasized the importance of *culturally sensitive and adapted services* to reach, understand and treat adolescents with asylum-seeker or refugee status. Most participants used concepts such as ‘cultural competence’ and ‘cultural sensitivity’ to describe strategies to manage challenges of cultural diversity in various clinical encounters. However, despite recognising the importance of culture, it was not always clear how healthcare professionals practiced cultural competence and cultural sensitivity. Moreover, cultural diversity concepts are often blurry, value-based and politically correct answers to improve mental health services for minority groups. Conceptual analysis and critique of cultural competence have been recommended to: ‘improve the cultural responsiveness, appropriateness and effectiveness of clinical services, and in doing so contribute to reducing health disparities’ (Kirmayer, 2012, p. 149). In recent decades, intense debate around cultural competence has generated important discussions about the relevance and ability to address systemic problems in mental health services, and to achieve health equity. Concepts such as ‘cultural awareness’, ‘cultural humility’, ‘cultural appropriateness’ and ‘cultural safety’ have been developed and discussed (Danso, 2018). ‘Cultural awareness’ refers to how other cultures influence thoughts and behaviours, respecting other cultures, and reflecting on one's own beliefs and values (Danso, 2018). ‘Cultural humility’ emphasizes understanding personal and others’ perspectives, acknowledging own prejudices and misperceptions, and addressing power imbalance in healthcare. It requires professionals to learn directly from their patients. Our study reflected such a view, in which healthcare professionals emphasized the importance of understanding their own culture, and learn about their patients’ cultures. ‘Culturally appropriate services’ recognize the unique characters of minority groups and any suppression they have been exposed to, and are adapted to their cultural contexts (Danso, 2018), whereas ‘culturally safe services’ create environments in which individuals feel safe expressing their cultural identity (Williams, 1999). Creating ‘culturally safe services’ requires space for participants to identify and evaluate their beliefs and values, how it may impact others, and address relational inequalities. Healthcare professionals should reflect on their own cultural identity and how this impacts their practice, ensuring everyone feels heard and respected (Richardson & Williams, 2007). This approach supports cultural identity and promotes personal empowerment.

Existing literature offers various overlapping concepts that highlight the importance of cultural variety and helps tailor health services for minority youth, including asylum seekers and refugees. Regardless of healthcare professionals’ and health policymakers’ preferred terminology, we argue for a systematic, reflexive approach at a systems level where definitions and practices are continuously discussed and developed within institutional contexts. This approach requires healthcare professionals to share experiences across primary and secondary care settings, while also incorporating the perspectives of service users and their families. This approach could inform education programmes across professions, guide development of national practice standards, and evidence-based policymaking. Norway and other welfare states are increasingly diverse, requiring engagement with heterogeneous populations (Jones et al., 2021; Winkler, 2016), including adolescents from various cultural backgrounds. At the individual level, healthcare professionals should reflect on how their cultural backgrounds influence their perceptions. Promoting this awareness using tools, training and integration into basic and postgraduate education, can inform policy development.

Although healthcare professionals’ openness to exploring their own and others’ cultural backgrounds can be considered instrumental to provide culturally appropriate and safe mental health services, having fixed values, beliefs and practices prevalent within different ethnic groups may be understood as essentialist (Williamson & Harrison, 2010). This approach suggests that cultural education during basic or postgraduate training may be sufficient to prepare healthcare professionals to care for youth with asylum-seeker and refugee backgrounds. However, this perspective assumes that culture remains largely static and unchanging. Blanchet Garneau and Pepin (2015) propose a constructivist perspective of culture as socially constructed and influenced by multiple contextual factors, including social, political and economic influences. This perspective recognizes culture as a social phenomenon, in which individuals from the same ethnic group can differ significantly and that cultural identity may change over time. It therefore prevents stereotypical thinking about youth from similar backgrounds. Some healthcare professionals in our study were aware of this perspective. As one noted, identities may vary across contexts that contribute to ‘define’ a person. More extensive explorations of healthcare professionals’ understandings of culture, including as a social phenomenon, could help to better describe their understandings of cultural competence and inform basic and postgraduate training programmes.

According to Blanchet Garneau and Pepin (2015), a constructivist understanding of cultural competence is developed in partnership with youth, families and communities – which aligns with the *user involvement* theme in our study. Healthcare professionals emphasized the importance of focusing on adolescents’ needs, allowing them to guide therapy and participate in decision-making. Shared decision-making is an increasingly used concept in youth mental health and represents a key aspect of collaboration in which ‘two autonomous and uncoerced agents both commit to actions that neither has reason to want to change based on their understanding of anticipated outcomes given the situation at hand and of the intended actions of the other party’ (Chambers, 2022, p. 1). Research suggests that shared decision-making may empower adolescents (Hayes et al., 2019), increase their engagement in treatment and reduce drop-out rates (Bjønness et al., 2020; Hart et al., 2005; Ranney et al., 2015). However, the outcomes of user involvement for adolescents with asylum-seeker and refugee backgrounds remains unexplored (Viksveen et al., 2022). Although healthcare professionals in our study emphasized the importance of involving adolescents in their mental healthcare, little attention was given to families, although family involvement is more common in minority groups, particularly within collectivist cultures (Fa’alogo-Lilo & Cartwright, 2021; Snowden, 2007). Individuals from collectivist cultures often see health (and mental health) differently, emphasizing interdependence and family involvement (Sundararajan et al., 2013). In some cases they may play significant roles in patients’ management of distress (Chase & Sapkota, 2017). Refugees typically come from collectivistic cultures that value family connection and involvement (Mak & Wieling, 2022). Culturally appropriate, effective and safe ways of involving social networks, especially families, in these adolescents’ mental healthcare should therefore be further explored. This requires knowledge about diverse understandings, because immigrant parents may struggle to identify mental health disorders and can contribute to underuse of services (Verhulp et al., 2013; Wang et al., 2019). Raising awareness among parents and broader networks is therefore important.

Healthcare professionals in our study emphasized the importance of *low threshold and outreach services* to improve use of mental health services. They acknowledged the stigma surrounding mental health among adolescents with asylum-seeker and refugee backgrounds, because they lack familiarity with the Norwegian healthcare system. Professionals recommended that these adolescents should be able to contact specialist health services directly, without a referral. Moreover, they suggested offering more than the standard ten sessions and recommended outreach services in safe environments like schools or homes. Their suggestions surpassed current boundaries in Norway. Participants noted that adolescents should have easy access to school nurses, who may provide mental health information, flexible referral routines and referral to secondary/specialist care when needed. The role of schools has also been emphasized by others. Fazel et al. (2016) emphasized the potential role of teachers in establishing contact with mental health services, a role that was not addressed in our study. However, participants recommended extending existing legislation by allowing adolescents under 16 to seek help without parental involvement.

The final theme in our study, *building a trusting relationship*, emphasized a strong therapeutic relationship, which has been widely recognized internationally to achieve positive outcomes in mental healthcare, irrespective of ethnic background. Healthcare professionals in our study considered trust as more important for asylum-seeker and refugee adolescents, given their traumatic experiences and potential scepticism towards strangers. A striking empirical example was the challenge of managing language barriers in care. Many adolescents feared that private information could be misunderstood or misused by healthcare professionals or interpreters. Some, including interpreters with traumatic experiences, were unfamiliar with concepts like mental health or mental illness. Kidron and Kirmayer (2019) highlight dilemmas of linking idioms of distress to co-morbid illness constructs and describe a paradox of culture-sensitive pathologization of distress. Healthcare professionals may engage with and assess asylum-seeking and refugee youth differently than their majority population peers. A recent Norwegian study found that general practitioners were more likely to diagnose refugees with PTSD than Norwegian patients, despite identical symptoms (Harris et al., 2021). From our perspective, building trusting relationships is essential to avoid inappropriate pathologization. We apply a sociological definition of trust, as the belief in another party's trust, honesty, fairness or benevolence, referring here to trust in healthcare professionals and the public health system (Sztompka, 1999). Participants emphasized that building trusting relationships with asylum-seeking and refugee adolescents requires knowledge, genuine interest, time and continuity of care. Trust in western healthcare is often lower among minority groups (Gilbert et al., 2019).

Healthcare professionals in this study emphasized the role of interpreters to overcome language barriers, foster understanding and build trust with asylum-seeking and refugee youth. Some recommended cultural interpreters, individuals familiar with both adolescents’ and professionals’ cultures, to clarify differences in understandings and support communication and facilitate mutual expectations (Abdi et al., 2020; Gómez & Pinazo, 2018). Interpreters should possess good linguistic and intercultural communication skills, together with domain-specific medical knowledge to ensure accurate translation (Abdi et al., 2020; Karwacka, 2014; Montalt-Resurrecció & Shuttleworth, 2012).

### Strengths and Limitations

The study's main limitation is the small sample size, which limits the transferability of the results across different samples and healthcare contexts. Results depend on the recruited participants (Salmon, 2013), and the researchers’ interpretations. Although participants were purposefully selected to reflect diverse professional backgrounds, increasing the relevance to other mental health professionals working with refugee and asylum-seeking youth, the impact of gender imbalance with the limited number of male participants remains unclear. Transferability to other age groups and other healthcare settings is also uncertain.

Measures were taken to strengthen the trustworthiness of the research (Lincoln & Guba, 1985; Rodwell & Byers, 1997), including an inductive, exploratory approach based on qualitative interviews (Guest et al., 2012). Researchers’ varied backgrounds enriched data analysis and interpretation, limiting undue influence by singular researchers (Wengraf, 2001). Semi-structured interviews followed predetermined interview guides, focusing on two main open-ended questions, with participants encouraged to share freely. Follow-up questions clarified and expanded responses, although some concepts, like cultural competence and sensitivity, were not always understood, warranting further exploration. Verbatim transcriptions preserved participant authenticity and enabled transparent reporting of direct quotes. Data was repeatedly reviewed to ensure accurate representation of participant experiences, and the richness of responses and clarity of the different research stages further support the study's credibility and confirmability.

### Future Recommendations

We are putting forward recommendations based on the current study, supplemented with findings from the existing research literature in the field of mental health service provision for adolescents who have a background as asylum seekers and refugees. This was a small qualitative study and the existing literature is not exhaustive in describing all types of experiences from clinical practice. Hence, we are not suggesting the following recommendations as definitive, but they are meant as contributions to the practice field based on the existing research literature. We suggest that national and possibly international guidelines should be developed to serve as recommendations for the development and provision of healthcare services that better can meet the needs of adolescents who have a background as asylum seekers and refugees.

First, we suggest there is a need to develop training both in basic education for health professionals, as well as ongoing postgraduate training to update and ensure awareness of and knowledge about challenges and possible solutions for how to engage in dialogue with these groups of youth. Training should be cross- and inter-professional; i.e. made available for persons with different professional backgrounds and to integrate experiences from different clinical contexts. It should also contribute to facilitate understanding of the meaning of and reflection about different concepts that are helpful to this practice field, such as cultural awareness, cultural humility and cultural safety. A constructivist approach to cultural competence training may be suitable to enable healthcare professionals to understand the dynamic nature of culture. Moreover, training should be practice-based by addressing concrete challenges healthcare professionals face in everyday meetings with adolescents and their families, possibly by providing opportunities to reflect on clinical experiences and through hands-on simulation exercises to train healthcare professionals’ skills in meeting challenges in effective and culturally sensitive ways.

Second, a systematic, reflexive approach to increase cultural competence should include ongoing discussions and development of terminology and practices; sharing of experiences; and integration of service users’ and their families’ perspectives, as well as organizations representing the interests of and providing support for persons with refugee backgrounds. Such an approach could possibly be carried out under the guidance of researchers and clinicians with experience from this practice field. Moreover, personnel responsible for organizing the healthcare services should be included to raise their awareness of the challenges faced in everyday clinical practices, so they may be better equipped to facilitate changes. Reflexive work may be considered to be complex and time-consuming, but useful models and guidelines have been developed for nurses and other healthcare professionals to support such processes in practice (Koshy et al., 2017; Rolfe et al., 2001).

This leads to our third recommendation, to provide low threshold services that are easily accessible to adolescents. Information about the existing services must be made easily available; for example, by ensuring that health classes, which are currently provided by nurses in Norwegian schools, are best adapted to also meet the needs of adolescents with a background as refugees and asylum seekers. The current standardized healthcare system requiring referral from primary to secondary care practitioners does according to the study participants serve as a culturally insensitive barrier preventing many adolescents with asylum-seeker and refugee backgrounds to seek and receive help from the healthcare services. This could include the possibility for others (e.g. family members, teachers, sports coaches) to ‘refer’ youth to the services, as well as an option to ‘self-refer’, for an initial assessment and potentially treatment provided by healthcare professionals.

Fourth, measures must be taken to develop a trusting relationship between adolescents and healthcare professionals. This requires learning to know each individual adolescent, their background and their understanding of their health and life situation, at a pace they feel comfortable with. There is therefore a need for continuity of services beyond the current standardized system available in Norway within secondary care services, as trust is built over time. Adolescents must experience healthcare providers as someone who is reliable and who is there for them. Facilitation of understanding and building of trust are also linked to the provision of professional interpreters who can help overcome language barriers. These interpreters must have sufficient training, including good language skills, sufficient knowledge of the field of healthcare, knowledge about ethics and training for ethically sound practice, and cultural competence to enable understanding of adolescents’ behaviour, descriptions and contexts.

Following the current study, a number of additional topics could be considered for further research. It would be helpful to gain additional insights into the knowledge and needs of healthcare professionals when working with adolescents who have a background as asylum seekers and refugees to contribute to set up and further develop education and training programmes for professionals. Also, insights into the needs of adolescents – both at the individual (for their own care) and systems level (for contributions to development of services) – depending on their age, gender, educational backgrounds, socio-economic status, ethnic background and religious beliefs would provide further insights that could be helpful in service provision. Further identification of additional barriers to service use and alternatives to clinic-based treatment and support could also be explored. An example is investigation of low threshold services including outreach services to meet adolescents in local environments where they feel safe for those who are reluctant to seek help within the established services. Ways of resolving challenges arising from differences in perspectives and interests arising between adolescents and their families would be helpful. Further approaches to reduce the impact of stigma in mental health and associated with use of mental health services are also wanted. Another research topic is safety in healthcare services for adolescents who are at risk of self-harm or harming others. The effectiveness and cost-effectiveness of services should also be assessed, both to ensure that services are as effective as possible and in light of financial constraints that exist within healthcare services in most countries. Finally, an exploration of involvement of adolescents with refugee backgrounds in research could be beneficial to further improve the research field.

## Conclusion

This study helped to explore healthcare professionals’ perspectives to provide solutions to overcome some barriers to mental health service use among adolescents who have a background as asylum seekers or refugees. Culturally sensitive and adapted services where healthcare professionals possess cultural competence and behave with cultural humility may help to build a trusting relationship, reduce mental health stigma and support adolescents to feel safe to express themselves. This requires healthcare professionals to be aware of their own cultural background, learn about other cultures, and to engage with adolescents’ values and beliefs. User involvement is central to meet adolescents’ mental health needs. Education and training are required to enable healthcare professionals to develop sufficient cultural competence. Low threshold and outreach services may facilitate help-seeking. Moreover, skilled interpreters, including cultural interpreters, play an important role in facilitating culturally safe health services to meet the mental health needs of adolescents with a background as asylum seekers and refugees.

## Appendix A: Interview guide

**What are healthcare professionals’ perspectives on what it takes for adolescents with status as asylum seekers or refugees to seek professional help for mental health problems and to feel they benefit from the help?**

**A qualitative study for improvement of the healthcare services.**

**1. What do you think is important in order for adolescents with status as asylum seekers or refugees to seek professional health for mental health conditions and challenges?**
  A. How do adolescents “find their way” to you as a healthcare professional?
  B. Which factors do you think are most important in order for adolescents to choose to seek help?
  C. What are the most important barriers for their help seeking?
  D. Which role do you think culture plays with regards to adolescents’ choice to seek help?
  E. Is there something you would recommend in order to encourage these youth to seek help with healthcare professionals when they need it?
**2. What do think is important in order for adolescents to feel they benefit from the help?**
  A. What do they say about what type of help they want or need?
  B. Which role does trust play? For them to feel welcome? Understood? Safe?
  C. What to you think helps youth to speak openly and freely about their mental health?
  D. What impression do you have of adolescents’ relationship to the term “mental health”? And how does culture influence this?
  E. Which roles do you think user involvement and shared decision-making play?
  F. Which experiences do you have with using interpreters?
